# PD1+ T Regulatory Cells Are Not Sufficient to Protect from Gestational Hypertension

**DOI:** 10.3390/ijms26072860

**Published:** 2025-03-21

**Authors:** Martyna Tomaszewicz, Katarzyna Stefańska, Joanna Dębska-Zielkowska, Dorota Zamkowska, Karolina Piekarska, Bogusław Tymoniuk, Przemysław Adamski, Joanna Jassem-Bobowicz, Dorota Madej, Piotr Trzonkowski, Natalia Maria Marek-Trzonkowska, Maciej Zieliński

**Affiliations:** 1 Department of Medical Immunology, Faculty of Medicine, Medical University of Gdańsk, 80-210 Gdańsk, Poland; martyna.tomaszewicz@gumed.edu.pl (M.T.); ptrzon@gumed.edu.pl (P.T.); 2 PolTREG S.A., 80-298 Gdańsk, Poland; 3 Department of Gynecology and Obstetrics, Faculty of Medicine, Medical University of Gdańsk, 80-210 Gdańsk, Poland; 4 Gameta Gdynia Center of Health, 81-347 Gdynia, Poland; 5 Department of Immunology and Allergy, Medical University of Lódź, 92-213 Lódź, Poland; 6 Division of Neonatology, Faculty of Medicine, Medical University of Gdańsk, 80-210 Gdańsk, Poland; 7 International Centre for Cancer Vaccine Science Cancer Immunology Group, University of Gdańsk, 80-822 Gdańsk, Poland; 8 Laboratory of Immunoregulation and Cellular Therapies, Department of Family Medicine, Medical University of Gdańsk, 80-211 Gdańsk, Poland

**Keywords:** preeclampsia, gestational hypertension, immunology, regulatory T cells, PD-1

## Abstract

Tolerance to foetal tissues in pregnancy depends on the match between mother and child. CD4+Foxp3+ regulatory T cells (Tregs), which are involved in peripheral tolerance, may facilitate this effect. Previous findings have indicated that the number of missing KIR ligands (MSLs) between mother and child correlates with the risk of gestational hypertension (GH) and preeclampsia (PE). This study tested whether Tregs are involved in the pathogenesis of gestational disorders. In total, 57 pregnant women participated, including 39 with hypertensive disorders of pregnancy and 18 healthy controls. Treg phenotypes were evaluated using multicolour flow cytometry. Killer cell immunoglobulin-like receptors (KIRs) and their ligands were assessed using NGS and PCR-SSO typing. The correlation between the MSLs and Treg antigen expression was evaluated. The pregnancy-related hypertensive groups differ from the healthy control group in the frequency of particular Treg subsets. However, there was a correlation between an increasing number of MSLs and only one subset of Tregs, which was PD-1+ Tregs. Surprisingly, women suffering from GH or PE had a significantly higher percentage of PD-1+ Tregs than healthy pregnant women. The percentages of several other populations of Tregs, such as those expressing CCR4, CCR10, CD39, and CD73, were higher in healthy pregnant women than in those with GH or PE, but these numbers did not correlate with MSLs. The exhausted PD-1+ Treg cell subsets may play a crucial role in the pathogenesis of hypertensive disorders of pregnancy. It is also hypothesised that MSLrelated mechanisms trigger PD-1+ Treg expansion, but their increased number fails to provide protection against hypertensive conditions of pregnancy.

## 1. Introduction

Preeclampsia (PE) has long been considered a vascular complication of pregnancy. However, many other components, including immunological factors, have begun to clarify the disorder’s pathomechanism and risk factors.

Regulatory T cells (Tregs) are CD4+ CD25+ T cells with characteristic expression of transcription factor forkhead box P3 (Foxp3). This specific subpopulation is responsible for maintaining immune homeostasis by suppressing overreactive effector cells that target foreign or self-tissues. Although Treg dysfunction is a component of many autoimmune diseases, over time, Tregs have been shown to play a vital role in many other diseases, even those not directly related to the immune system [1].

Without a doubt, Tregs play a pivotal role in maintaining healthy pregnancies. From the moment of extravillous trophoblast implantation in the uterus and early pregnancy, infiltrating Tregs and Th1 cells inhibit Th2 and Th17 responses, establishing a sustainable environment where the processes of silencing and inducing inflammation are well balanced [2]. During this early phase, Tregs, in balanced cooperation with natural killer (NK) cells, dendritic cells (DCs) and macrophages, facilitate trophoblast invasion to the uterus and placentation [3]. As pregnancy progresses into the mid and late stages, the number of foetal-antigen-induced Tregs systematically increases, preventing T and NK cell activation and enabling maintenance of pregnancy [4].

In humans, Tregs can be identified and subdivided into three major groups based on the expression of intracellular Foxp3 and surface CD45RA antigens. These groups include resting Tregs (naive Tregs, nTregs, CD45RAhighFoxp3low), cytokine Tregs (non-Tregs, exTregs, CD45RA-Foxp3low), and activated Tregs (effector Tregs, eTregs, CD45RA-Foxp3high) [5]. Sakaguchi et al. suggested that the proportion of these subpopulations may vary in healthy individuals and those with immune-dependent diseases [6].

The importance of Tregs’ functionality lies in the signals transduced through characteristic receptors called immune checkpoint molecules (e.g., cytotoxic T lymphocyte antigen 4 (CTLA-4) and programmed cell death protein 1 (PD-1 or CD279). Binding to specific ligands enables modulation of the immune response through the inhibition of cell-mediated inflammation [3]. PD-1 is one of the immune checkpoint molecules that is crucial in establishing immune homeostasis and tolerance [7,8]. The PD-1/programmed death ligand 1 (PD-L1) pathway regulates T effector cell function and marks cell exhaustion [7,8]. Research has demonstrated the importance of blocking this pathway in enhancing antitumor responses [8] and its significance in monitoring the treatment of autoimmune diseases [9]. The influence of immune checkpoint molecules in pregnancy has not yet been fully elucidated.

However, immunohistochemical staining with anti-PD-L1 antibodies has revealed the presence of PD-1 ligands on syncytiotrophoblast, the outer layer of the placenta that remains in direct contact with maternal blood [10,11,12].

The PD-1/PD-L1 interaction forces T cell regulation by suppressing T cell activation. Thus, blocking with anti-PD-1 and anti-PD-L1 antibodies induces Treg suppression, promoting a proinflammatory milieu and, consequently, causing pregnancy losses [2,3,10]. Research has also shown a reduced number and exhausted PD-1+ phenotype of Tregs in preeclamptic women [13,14,15,16]. This could be a key aspect of the significance of the PD-1/PD-L1 pathway in promoting maternal–foetal tolerance utilising immune mechanisms.

The key factor in energy production during placental implantation and development is mitochondrial activity [17]. The process ultimately stores energy in adenosine-5′-triphosphate (ATP) [18]. The energy from the extracellular pool of ATP is later released in a two-step process. First, ectonucleoside triphosphate phosphohydrolase 1 (CD39) converts ATP into adenosine-5′-monophosphate (AMP). Then, ecto-5′-nucleotidase (CD73) dephosphorylates AMP into adenosine (ADO) [19].

Under physiological conditions in humans, the extracellular ATP (eATP) level is relatively low but can rise multiple times in cell injury, stress, or necrosis [20]. There are indications that an elevated level of extracellular ATP may become a ‘danger’ signal, known as a danger-associated molecular pattern (DAMP) acting through interaction with purinergic receptors. The above mechanism is likely to be involved in the aetiology of gestational hypertensive disorders in which elevated ATP levels trigger inflammatory responses that exacerbate PE symptoms and, consequently, damage the placenta and inhibit hemopexin activity, which is crucial for blood pressure regulation [20,21]. In vitro stimulation of T cells with ATP has resulted in T cell activation, differentiation into proinflammatory Th17 cells and production of IL-2 and INF-γ [22,23].

Human leukocyte antigens (HLAs) are crucial for distinguishing between self and foreign antigens. Multiple sources have indicated that HLA antigens play a significant role in pregnancy and may also be important in the development of PE [24,25]. The initial stages of embryo development, specifically the villous cytotrophoblasts and syncytiotrophoblasts, do not express HLA class I and II antigens which may serve to protect them from T cell alloreactivity [2]. Subsequently, invasive extravillous trophoblasts begin to express HLA-C, -G, -E and -F.

HLA-G and HLA-E molecules are highly expressed in the extravillous trophoblast cells of the placenta and play a vital role in creating the maternal–foetal tolerance barrier [26]. HLA-G interactions with killer cell immunoglobulin-like receptors (KIRs) expressed on NK cells protect cells from NK cell-mediated lysis [27]. Tersigini et al. demonstrated that HLA-DR (Class II HLA) was detected in the syncytiotrophoblast and syncytiotrophoblast-derived extracellular vesicles of patients with PE but not in healthy control cases. Here, atypical expression and subsequent inadequate maternal immunotolerance are proposed as an immunogenic trigger of PE occurrence [28]. In our previous research, we highlighted that the KIR–HLA interaction and subsequent inhibition or activation of NK cells are crucial in the pathomechanism of hypertensive disorders of pregnancy, as NK cells of mothers with hypertensive disorders of pregnancy were found to be more cytotoxic than in healthy cases [24].

Given that variations have been identified in the quantity and functionality of Tregs in healthy versus complicated pregnancies, we aimed to identify differences in the Treg phenotype to assess its potential influence on the pathogenesis of gestational hypertension (GH) and PE compared to healthy pregnancies. Additionally, we wanted to investigate whether the number of missing KIR ligands (MSLs) in mother and child relationships influences Treg phenotype, possibly affecting their function in pregnancy-related disorders.

## 2. Results

### 2.1. Major Findings

We found that the pregnancy-related hypertensive groups differed from the healthy control (HC) group in terms of exTreg, nTreg CCR4+, nTreg CCR10+, nTreg CD39+, nTreg CD73+, eTreg CD73+, eTreg CD152+, Foxp3+Helios- CD279+, Foxp3+Helios+ CD279+ and Foxp3-Helios+ CD279+ populations.

Moreover, by analysing the dependence of the Treg phenotype MSL quantity, we found significant differences in the eTreg CD279, exTreg CD279, Foxp3Foxp3+Helios- CD279 and Foxp3+Helios+ CD279 percentages.

### 2.2. Missing KIR Ligands Correlation with the Frequency of Treg Subpopulations

**Figure 1:**
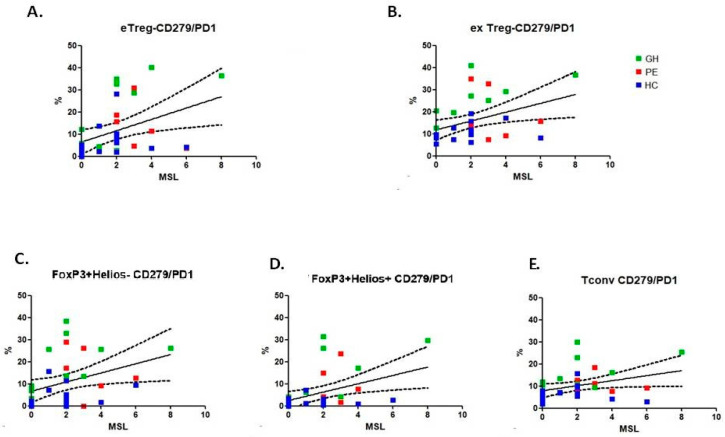
Association between the number of missing KIR ligands (MSLs) and the percentage of T regulatory/T conventional expressing PD-1. The association analysis included the whole group–i.e., the preeclampsia (PE), gestational hypertension (GH) and healthy control (HC) groups. The red, green and blue points correspond to PE, GH and HC groups, respectively. The Spearman’s test *p* < 0.05 is considered significant. (**A**): r = 0.4416, *p* = 0.0089; (**B**): r = 0.4081, *p* = 0.0166; (**C**): r = 0.4472, *p* = 0.0088; (**D**): r = 0.4418, *p* = 0.0089; (**E**): r = 0.3638, *p* = 0.0344.

The absolute number of MSL was associated with the frequency of PD-1 (CD279)-expressing eTregs (Figure 1A), exTregs (Figure 1B), Foxp3+Helios- (Figure 1C), Foxp3+Helios+ (Figure 1D) and conventional T cells (Figure 1E).

### 2.3. Treg Phenotype

There were significant differences in the percentage of particular T cell subpopulations between the PE, GH and HC groups.

**Figure 2:**
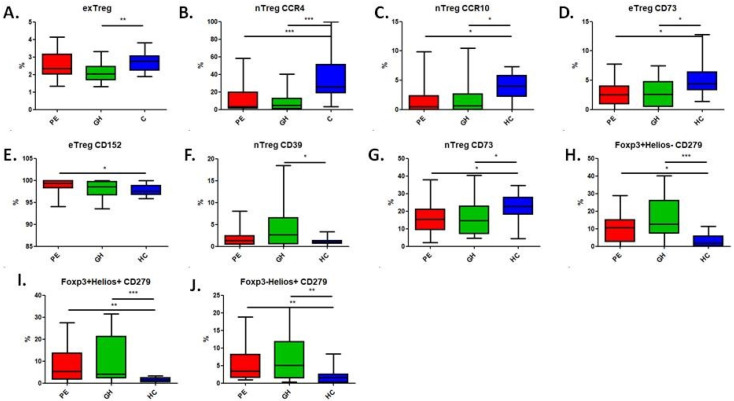
Percentage of cells expressing particular antigens: exTregs, nTregs, eTregs, Foxp3+Helios- Tregs, Foxp3+Helios+ Tregs, and Foxp3-Helios+ Tregs in the study groups: PE (red), GH (green) and HC (blue). Kruskal–Wallis with Dunn’s multiple comparison test. *p* < 0.05 is considered significant * *p* < 0.05; ** *p* < 0.01; *** *p* < 0.001. (**A**): *p* = 0.0039; (**B**): *p* < 0.001; (**C**): *p* = 0.0280; (**D**): *p* = 0.0145; (**E**): *p* = 0.0403; (**F**): *p* = 0.0465; (**G**): *p* = 0.0218; (**H**): *p* = 0.0013; (**I**): *p* = 0.0017; (**J**): *p* = 0.048.

The HC group was characterised by a higher frequency of exTreg (Figure 2A), nTreg CCR4 (Figure 2B), nTreg CCR10 (Figure 2C) and eTreg CD73 (Figure 2D) than the hypertensive groups.

Women with diagnosed PE showed a statistically significant increase in eTreg expressing CD152 (Figure 2E) compared to the HC group.

nTreg CD39+ was found at a higher percentage in GH patients than in HCs (Figure 2F).

nTreg expressing CD73 was found at a higher percentage in healthy women than in the hypertensive groups (Figure 2G).

Patients suffering from hypertension showed higher percentages of Tregs expressing PD-1 on the surface than the HC group: Foxp3+Helios-CD279+ Tregs (Figure 2H), Foxp3+Helios+CD279+ Tregs (Figure 2I) and Foxp3-Helios+CD279+ Tregs (Figure 2J).

**Figure 3:**
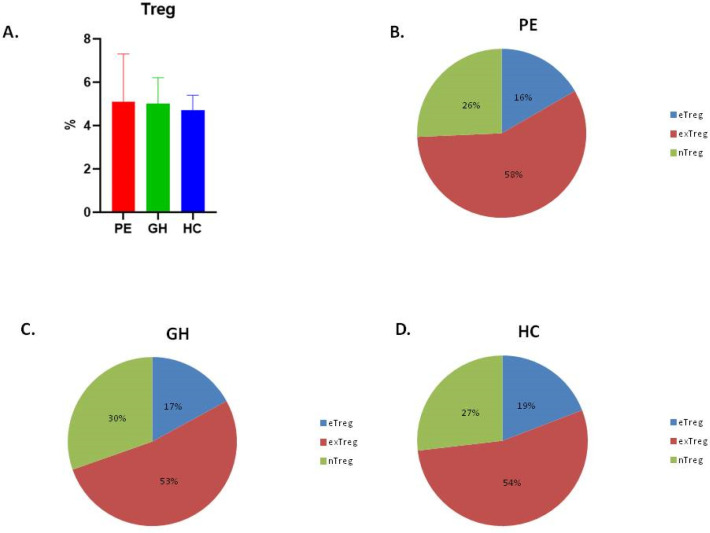
Percentages of Tregs cells in peripheral blood of PE (red), GH (green) and HC (blue) patients (**A**). Kruskal–Wallis with Dunn’s multiple comparison test, *p* = 0.2911. The mean percentages of nTreg (green), eTreg (blue) and exTreg (red) within Foxp3 positive cells in PE (**B**), GH (**C**) and HC (**D**) groups are shown.

There were no significant alterations in Treg percentages in the peripheral blood of the PE, GH and HC patients (Figure 3A). Within Tregs, eTreg, nTreg and exTreg populations did not differ between the PE, GH and HC groups (Figure 3B–D).

**Figure 4:**
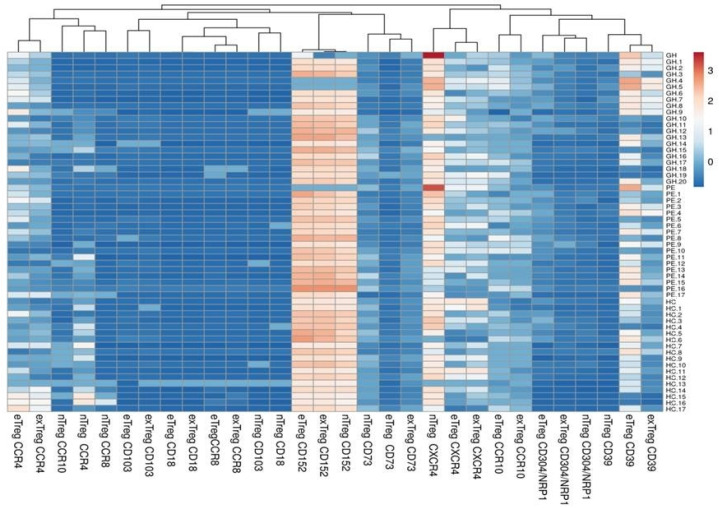
Heatmap analysis of nTreg, eTreg, and exTreg expression in three groups: preeclampsia (PE 0–18), gestational hypertension (GH 0–20), and healthy control (HC 0–17).

The percentages of nTreg, eTreg and exTreg (expressing CCR4, CCR8, CCR10, CD103, CD18, CD152, CD73, CXCR4, CD304 and CD39) were screened using a heatmap approach to determine whether there were any significant parameters for PE or GH patients compared to HCs (Figure 4). No significant relationships were found among the groups.

### 2.4. Receiver Operating Characteristics

**Figure 5:**
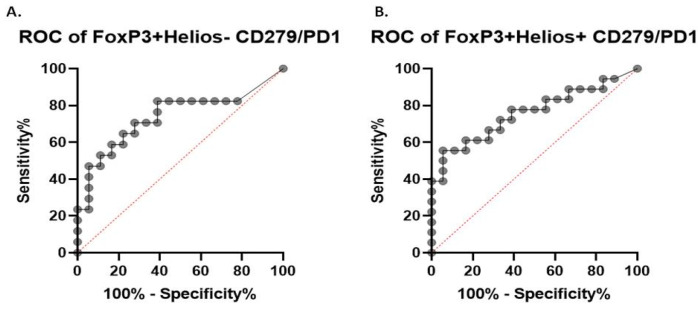
ROC analysis of the PE group versus the HC group. *p* < 0.05 is considered significant (line with grey points-ROC curve, red line-line of identity, baseline).

**Figure 6:**
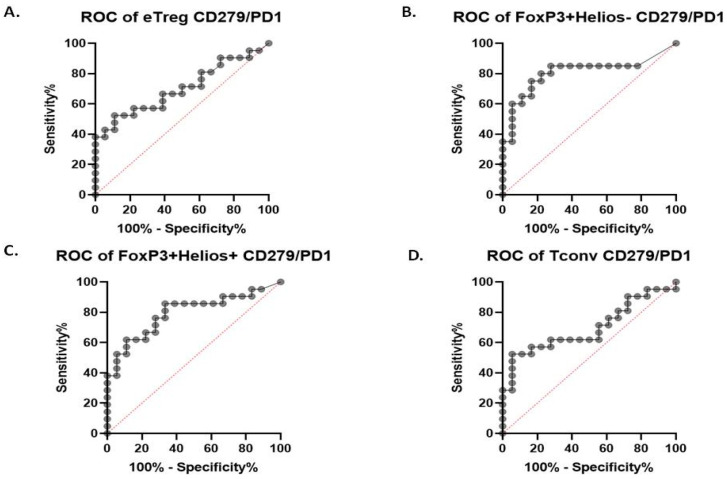
ROC analysis of the GH group versus the HC group. *p* < 0.05 is considered significant (line with grey points-ROC curve, red line-line of identity, baseline).

The parameters that showed a correlation with the quantity of MSLs were included in the receiver operating characteristics (ROC) analysis (Table 1). Cut-off values were established to distinguish between the PE, GH and HC groups with the highest sensitivity and specificity (Figure 5 and Figure 6, respectively; Supplementary Data Table S1). The results of the entire analysis are presented in the Supplementary Data (Figures S2 and S3)

Furthermore, we conducted an ROC analysis for the PE and GH groups (Figure S4) to determine whether certain markers could differentiate PE from other hypertensive disorders of pregnancy, but the results were not statistically significant.

Detailed results are presented in the Supplementary Data (Table S1).

## 3. Discussion

The findings of Saito S. suggest that immune activation is restricted locally to the maternal–foetal interface [4]. The peripheral subpopulation of immune cells seems to reflect the local (decidual) changes, as evidenced by the increased level of PD-1+ cells in the decidua, which is also visible in the periphery [29]. Our research reveals differences in PD-1 percentages in Treg subpopulations between the PE/GH and HC groups. Additionally, we found correlations between MSL quantity and PD-1 expression on Treg subpopulations and T conventional cells in the whole group (PE, GH and HC in one analysis). The more MSLs present, the higher the expression of PD-1 on the cell. We demonstrated that elevated PD-1 (CD279) expression on specific T cell subsets could serve as a potential marker of pathological changes in pregnancy possibly caused by an incompatibility between the mother and the child (MSL number). The key point is that the PD-1 antigen on Tregs reflects their suppressive potential against proinflammatory subsets. Therefore, activating this pathway may reduce inflammation in the maternal–foetal interface. PD-1/PD-L1 pathway induction might trigger a tolerogenic maternal–foetal environment by promoting proper Treg function and inhibiting proinflammatory T effector cells.

In our previous research, we discovered that HLA compatibility determines PE severity. The more mismatched the HLA eplets between the mother and foetus, the higher the likelihood of an uncomplicated pregnancy [30]. Additionally, our other study showed that patients with GH and PE had a higher number of MSLs compared to the control group [24]. This suggests that an increasing number of MSLs may be co-responsible for the development of hypertensive disorders during pregnancy. However, noting that PD-1 showed higher expression on Tregs in GH patients than in PE patients, immune checkpoint molecules expressed as a result of existing inconsistencies appear to be an insufficient response from Tregs trying to resolve the incompatibility between mother and child. Recent studies have indicated that the placenta of women with PE is characterised by reduced expression of PD-L1 compared to healthy pregnancies [11,12]. Here, the presented pathomechanism of PE is connected with the downregulation of the JAK2/STAT5 signalling pathway. Our results may align with the findings of Tian et al. and Mittelberger et al., providing evidence of these changes that can also be found peripherally. In our opinion, the decrease in PD-L1 on the placenta may be associated with a compensatory increase in the percentage of PD-1 expressing Tregs. Therefore, we propose the impairment of the PD-1/PD-L1 axis as part of the induction mechanism of hypertensive diseases during pregnancy.

Nonetheless, elevated PD-1 expression is a well-known marker of cell exhaustion, which may not serve as an insufficient protection marker but rather as a sign of cell exhaustion [31]. Therefore, in this scenario, PD-1+ Tregs may potentially be a reason for disease occurrence [13].

Elevated CD39 expression could indicate heightened eATP levels, leading to the need for metabolisation. However, in our study, this effect was only significant in nTreg and eTreg in patients with GH, not in patients with PE. In contrast to CD39, CD73, which is responsible for ADO generation, was decreased in both the PE and GH groups.

Given the vasodilatory, proangiogenic and immunomodulatory role of adenosine, it appears that ADO plays a substantial role in dynamic changes during pregnancy. Therefore, the imbalance of CD39 and CD73, particularly the decrease in CD73 in eTreg and nTreg of women with PE and GH observed in our research, may indicate another component of the pathomechanism of these disorders [23].

Another important observation is that the chemokine receptors CCR4 and CCR10 were decreased in hypertensive patients compared to HCs. This reduction may suggest defective cell migration of immunosuppressive cells into the decidua, leading to immune imbalance and abnormalities in vessel formation that could result in atypical implantation and the future development of pregnancy-related hypertension.

In this research, we proposed a set of antigens that could serve as markers for an increased risk of hypertensive pregnancy disorders. Additionally, we demonstrated changes in the phenotype of immune cells that may occur as a result of the pathomechanism of hypertensive diseases. We are aware that further investigation is needed to determine the exact mechanism behind these changes. However, to gain knowledge about the pathomechanism of placental dysfunction, it may be necessary to introduce more invasive methods to directly visualise the phenotype of the placenta-derived cells as well as changes to the placenta itself.

This proposal may represent a preliminary step towards recognising the significance of the immune system in the development of gestational hypertensive disorders.

## 4. Materials and Methods

### 4.1. Study Design

In this study, 57 women were enrolled between March 2018 and December 2019 at the Department of Obstetrics and Gynecology, Medical University of Gdańsk, Poland. Patients were between 27 and 42 weeks of gestation with a singleton pregnancy; 39 had symptoms of gestational hypertension or preeclampsia but no other co-morbidities.

Most of the patients were admitted after the 34th week of pregnancy. Patients admitted before the 32nd week of pregnancy were admitted due to exacerbation of hypertension, worsening of their general condition or foetal growth restriction (FGR).

The exclusion criteria are defined in the Supplementary Data (Supplementary Data S1. Study Exclusion Criteria).

This study was approved by the Bioethics Committee at the Medical University of Gdańsk (no. NKBBN/454/2014) and was conducted according to the principles of the Declaration of Helsinki. All participants provided written informed consent to participate in the study.

### 4.2. Patients

Based on the clinical and laboratory evaluations, according to the 2018 International Society for the Study of Hypertension in Pregnancy (ISSHP) guidelines [32], patients were classified into PE (*n* = 18), GH (*n* = 21) and HC (*n* = 18) groups. All patients were of Caucasian ethnicity.

The characteristics of the participants are presented in Table 2.

Hypertensive disorders related to pregnancy (e.g., PE and GH) were diagnosed when systolic blood pressure reached ≥ 140 mm Hg and diastolic blood pressure was 90 or above after 20 weeks of gestation in a previously normotensive pregnant woman without proteinuria or an indication of end-organ dysfunction. Detailed criteria for the diagnosis of PE are presented in the Supplementary Data (Supplementary Data S2. PE diagnosis).

**Figure 7:**
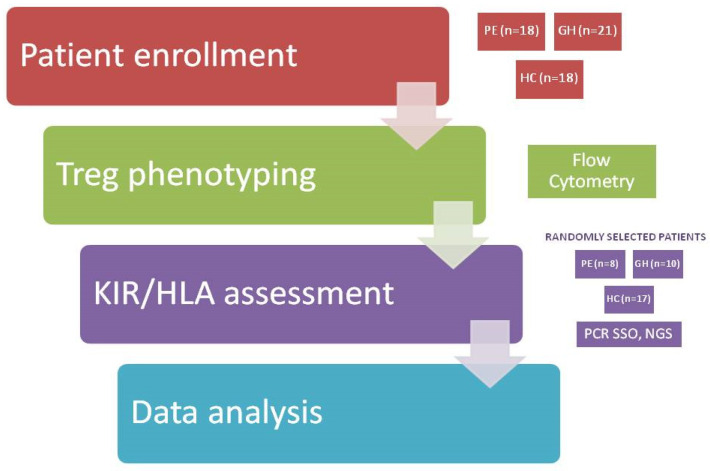
Study flow diagram.

A study flow diagram is provided in Figure 7.

### 4.3. Sampling

Blood samples were taken from patients with worsening hypertension symptoms during hospital admission. Blood samples from the control group were taken in the third trimester of gestation.

Oral swabs from neonates were taken at the obstetrics ward within two days of the birth.

Blood samples from mothers and swabs from neonates were used for the isolation of DNA to study KIR receptors and HLA antigens. The remaining blood samples from mothers were ficolled to obtain peripheral blood mononuclear cells (PBMCs) for flow cytometry. The sera from blood samples taken without anticoagulant were centrifuged and stored at −80 °C until use.

### 4.4. Regulatory T Cell Phenotype-Flow Cytometry

Ficoll–Paque density gradient centrifugation was used to isolate PBMCs from the blood samples. PBMCs were washed in phosphate-buffered saline (PBS), suspended in a small amount of PBS and stained using fluorochrome-conjugated monoclonal antibodies (mAbs) according to the manufacturer’s protocol. The protocol assumed the use of mAbs against surface antigens (clone) in the following combinations: CD3 (UCHT1), CD4 (SK3), CD25 (M-A251), CD127 (HIL-7R-M21), CD62L (DREG56), CD45RA (HI30), CD18 (L130), CCR8 (191704), CCR10 (314305), CD184 (1265), CD194 (161), CD103 (Ber ACT8), CD304 (12C2), CD39 (TU66), CD152 (BNI3), CD73 (AD2), CD279 (EH12.1), CD137 (4B4-1) and CD134 (ACT35) (Table 3).

Each of the above antibodies was used at the recommended volume. Cells were incubated with mAbs against cells’ surface antigens for 30 min at room temperature, washed in PBS and permeabilised with freshly prepared Foxp3/transcription factor fixation/permeabilization solution (Thermo Fisher Scientific, Waltham, MA, USA) for 60 min at 4 °C. After permeabilisation, the cells were washed in perm/wash solution and stained for intracellular CD152 (BNI3), Helios (22F6) and Foxp3 (236AIE7) for 30 min at 4 °C. In the next step, the cells were washed and suspended in PBS for flow cytometry analysis performed using an LSRFortessa Cell Analyzer (BD Bioscience, San Diego, CA, USA). The data were analysed using FACSDiva v9.0 software (BD Bioscience, San Diego, CA, USA) and Kaluza Analysis 2.3 Flow Cytometry software (Beckman Coulter, Brea, CA, USA).

The minimum backbone markers were used for gating purposes, which refers to CD3 and CD4. Cells were gated as demonstrated in the Supplementary Data (Supplementary Data Figure S1).

### 4.5. HLA and KIR Assessment

DNA isolation from the peripheral blood of mothers and from oral swabs from neonates was performed using a Sherlock AX kit (A&A Biotechnology, Gdańsk, Poland) according to the manufacturer’s protocol. HLA-A, HLA-B, HLA-C (class I) and HLA-DRB1, HLA-DQB1 (class II) were assessed using high-resolution next-generation sequencing (NGS) technologies in the Illumina MiniSeq System (MIA FORA NGS FLEX 5HLA Typing KIT, IMMUCOR). MIA FORA ver 1.0software was used to analyse and match the obtained sequences.

The LIFECODES KIR PCR-SSO (polymerase chain reaction-sequence specific oligonucleotide) Typing Kit (IMMUCOR, Norcross, GA, USA) was used in the KIR genotyping process which included 16 KIR genes: 2DS4 (in two variants: full length and deleted alleles), 3DP1, 2DP1, 3DS1, 3DL3, 3DL2, 3DL1, 2DS3, 2DS2, 2DS1, 2DL5, 2DL4, 2DL3, 2DL2, 2DL1 and 2DS5. The Luminex 200 flow platform was used to quantify the amplicons. The amplicons were analysed using the LIFECODES MATCH IT! DNA 1.3 software to generate KIR data.

MSLs refer to the absence of HLA epitopes (KIR ligands) in the foetus that correspond to the KIRs in the mother.

### 4.6. Statistics

As suggested by the data distribution, non-parametric statistics were used. Tests were conducted using Statistica 13 software (StatSoft, Kraków, Poland). The Kruskal–Wallis test evaluated differences among the Treg phenotypes in the PE, GH and HC groups. Additionally, the Kruskal–Wallis test was used to determine the dependence of MSL quantity (0, 1, ≥2) on Treg phenotypes. The Spearman’s test was applied to examine correlations between MSL quantity and Treg phenotypes.

The heatmap was generated using ClustVis (https://biit.cs.ut.ee/clustvis/, accessed on 12 July 2024) [33].

### 4.7. Study Limitations

Low number of patients in the control group: We are aware that the number of patients in the control group (*n* = 18) may limit the statistical significance of the findings; however, it was selected to closely match the quantity of the study group.

Statistically significant differences in maternal age: All of the patients were in the third trimester of pregnancy in which all the immunological parameters should be established on comparable levels. The percentage of PD-1 expressing Tregs was evaluated within the whole population of Tregs, and the number was not statistically different between study groups (Figure 3).

Study design: Research involving pregnant women is still treated with great care to ensure the welfare of both the mother and the fetus is not jeopardised. To acquire knowledge on the exact mechanism of placental dysfunction in pregnancy, it may be necessary to introduce more invasive diagnostic methods, which may compromise patient safety and could face resistance from bioethics committees. This could also be an issue in the later introduction into routine diagnostics.

## Figures and Tables

**Table 1:** Area under curve (AUC) results for statistically significant parameters. *p* < 0.05 is considered significant.

	AUC (95% CI)	*p*	Cut-Off (%)	Sensitivity (95% CI)	Specificity (95% CI)
PE vs. HC					
Foxp3+ Helios- CD279+	0.7386 [0.5645, 0.9126]	0.016	>10.12	52.94 (36.96–73.83)	88.89 (67.2–98.03)
Foxp3+ Helios+ CD279+	0.7593 [0.5978, 0.9208]	0.0079	>3.24	55.56 (33.72–75.44)	88.89 (67.2–98.03)
GH vs. HC					
eTreg CD279+	0.7011 [0.5362, 0.8659]	0.0323	>7.445	57.14 (36.55–75.53)	77.78 (54.79–91.0)
Foxp3+ Helios- CD279+	0.8056 [0.6560, 0.9551]	0.0013	>6.075	80 (58.4–91.93)	77.78 (54.79–91.0)
Foxp3+ Helios+ CD279+	0.7963 [0.6537, 0.9389]	0.0016	>2	85.71 (65.36–95.02)	66.67 (43.75–83.72)
Tconv CD279+	0.6958 [0.5280, 0.8635]	0.0371	>7.410	61.9 (40.88–79.25)	72.22 (49.13–87.5)

**Table 2:** Patient characteristics.

Parameter	PE (*n* = 18)	GH (*n* = 21)	C (*n* = 18)	*p* (Kruskal–Wallis Test)
Age [years]; mean ± SD	30.2 ± 5.1	30.7 ± 5.,1	31.1 ± 3.8	0.617
Length of gestation [weeks]; mean ± SD	34.6 ± 3.8	38.4 ± 2	39.8 ± 1.3	<0.0001
Body mass index [kg/m^2^]; median, min/max	30, 23/38	31, 24/41	26, 22/36	0.0007
Parity				
0	9	14	10	
1	6	2	6	
>1	3	5	2	

**Table 3:** Flow cytometry antigen panel.

Tube 1—Control	Tube 2—Basic Treg Phenotype	Tube 3—Chemokine Expression	Tube 4—Metabolome Analysis	Tube 5—Checkpoints
CD3 V500	CD3 V500	CD3 V500	CD3 V500	CD3 V500
CD4 PerCP	CD4 PerCP	CD4 Alexa700	CD4 PerCP	CD4 PerCP
CD25 PE	CD25 PE	CD25 BV786	CD25 PE	CD25 PE
CD127 FITC	CD127 FITC	CD127 BUV737	CD127 FITC	CD127 FITC
	CD45RA PECy7	CD45RA PECy7	CD45RA PECy7	CD45RA PECy7
	CD62L Alexa700	CD62L Alexa700	CD62L Alexa700	CD62L Alexa700
	Foxp3 APC	Foxp3 APC	Foxp3 APC	Foxp3 APC
	Helios eFluor450	Helios eFluor450	Helios eFluor450	Helios eFluor450
		CCR10 PE	CD73 BUV786	CD134/OX40 BV711
		CCR4 BV605	CD152 BV786	CD137/41BB BV650
		CCR8 PerCP	CD304/NRP1 Alexa700	CD274
		CD103 BUV395	CD39 BV650	CD279 BV605
		CD18 FITC		CD39 BUV737
		CXCR4 PE-CF594		

## Data Availability

Data is contained within the article and Supplementary Materials.

## Supplementary Materials

The following supporting information can be downloaded at: www.mdpi.com/article/10.3390/ijms26072860/s1. Refs [34,35,36] are cited in Supplementary Materials file.

## Abbreviations

ADO: adenosine; AMP: adenosine-5′-monophosphate; ATP: adenosine-5′-triphosphate; BMI: body mass index; CD: cluster of differentiation; CTLA-4: cytotoxic T lymphocyte antigen 4; DAMP: danger-associated molecular pattern; eATP: extracellular ATP; FGR: foetal growth restriction; Foxp3: forkhead box P3; GH: gestational hypertension; HC: healthy control, HLA: human leukocyte antigen; ISSHP: International Society for the Study of Hypertension in Pregnancy; KIR: killer cell immunoglobulin-like receptor; mAb: monoclonal antibody; MSL: missing KIR ligand; NK cell: natural killer cell; PBMC: peripheral blood mononuclear cell; PBS: phosphate-buffered saline; PD-1: programmed cell death protein 1; PD-L1: programmed death ligand 1; PE: preeclampsia; Treg: regulatory T cell.
