# Supplementary material for: PD1+ T Regulatory Cells Are Not Sufficient to Protect from Gestational Hypertension
Source: Int J Mol Sci. 2025 Mar 21;26(7):2860. doi: 10.3390/ijms26072860 (PMC11988647; doi:10.3390/ijms26072860)
Supplement: Supplementary file 1 [file ijms-26-02860-s001.zip › ijms-3465484-supplementary.pdf]

Supplementary Data

S1. Study exclusion criteria

Women with chronic secondary/essential hypertension, immunological diseases like Hashimoto's disease, diabetes mellitus, pre-existing renal disease, intrauterine fetal death, gestational diabetes, bacteriuria, multiple pregnancies, assisted reproductive technology in pregnancy, and premature rupture of membranes, were excluded. Patients with a BMI higher than 42 and those chronically administered aspirin or other anti-inflammatory agents were excluded.

S2. PE diagnosis

PE was diagnosed in patients with high blood pressure (24h blood pressure records) and new-onset proteinuria, i.e., when resting blood pressure was≥ 140/90 mmHg on two occasions that were at least 4h apart, and when significant proteinuria was detected in urine samples. Proteinuria was diagnosed with a urine protein/creatinine ratio (UPCR) ≥30 mg/dl cut-off value. [34,35]

In the absence of proteinuria, PE was diagnosed based on:

- hypertension in association with thrombocytopenia (platelet count <150,000/μL),
- impaired liver function (two-fold increase in blood levels of liver aminotransferases in comparison to the average concentration),
- a new development of renal insufficiency (elevated serum creatinine >1.02 mg/dL),
- pulmonary oedema, new-onset of cerebral or visual disturbances,
- uteroplacental dysfunction, including FGR. [34,36]

FGR was diagnosed as fetal abdominal circumference/estimated fetal weight <10th percentile combined with pulsatility index in the umbilical artery >95th percentile or pulsatility index in the uterine artery >95th percentile, or abdominal circumference/estimated fetal weight <3rd percentile, or absent end-diastolic flow in the umbilical artery. [35]

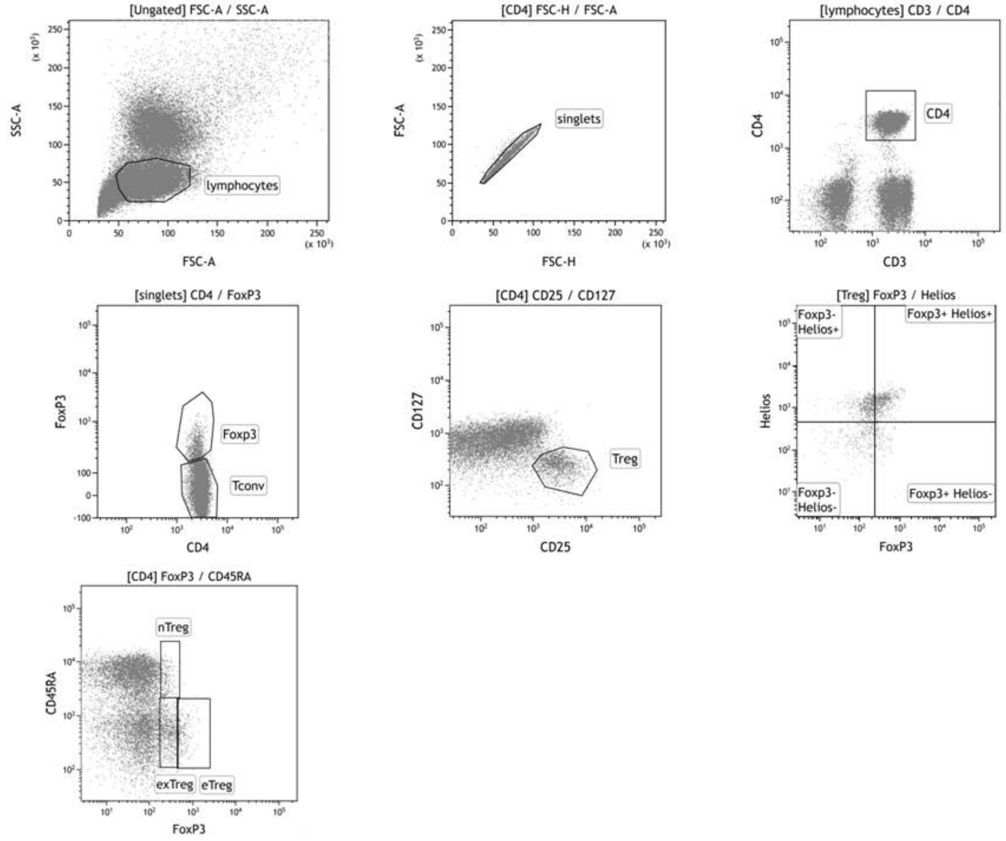

Figure S1. Gating strategy

T regulatory cells were gated as CD4+CD25+CD127- cells. In humans, three Treg subpopulations can be identified by CD45RA and intracellular Foxp3 expression: resting Treg CD45RA+Foxp3^{low} (nTreg), cytokine Treg CD45RA-FoxP3^{low} (exTreg), and activated Treg CD45-FoxP3^{high} (eTreg) [5]

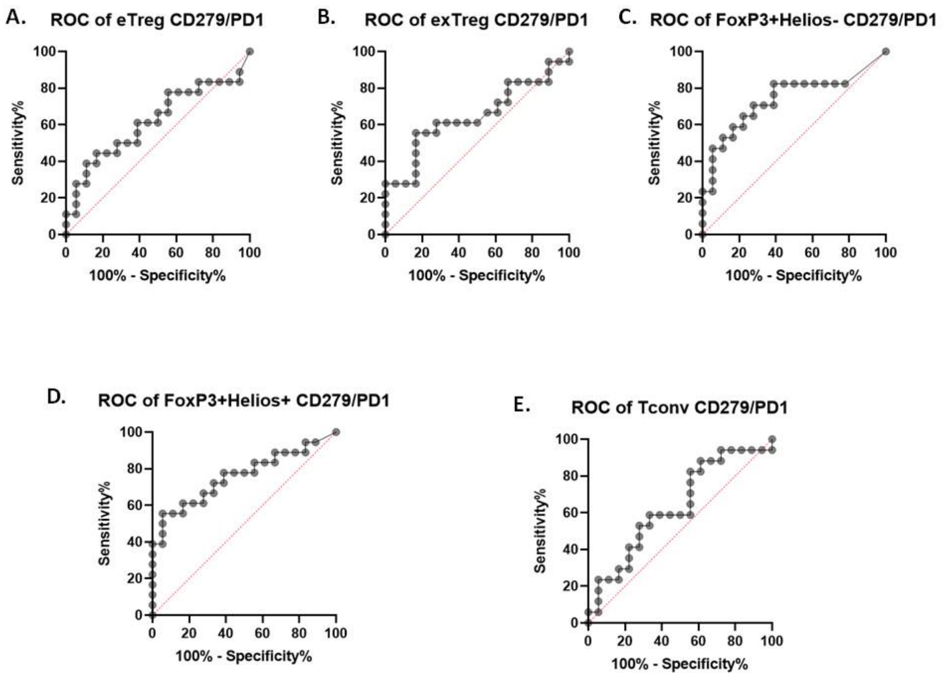

Figure S2. The ROC analysis of PE versus HC group.

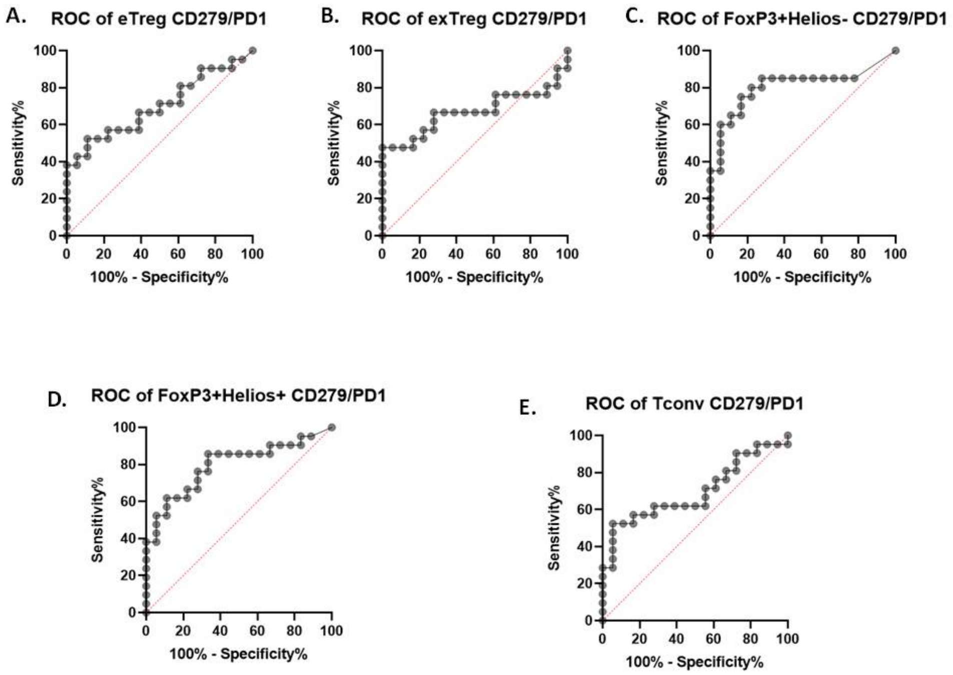

Figure S3. The ROC analysis of GH versus HC group.

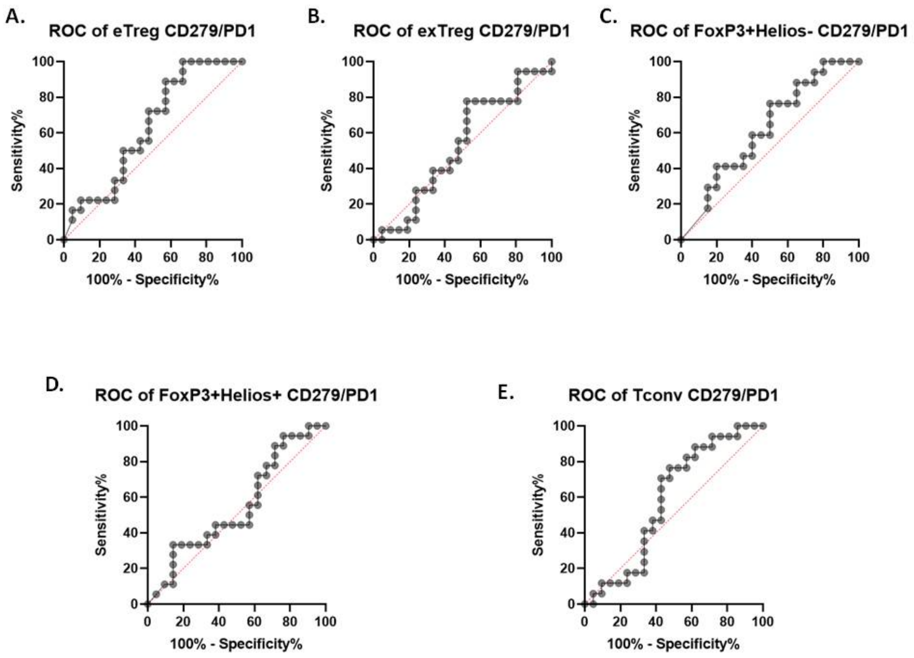

Figure S4. The ROC analysis of PE versus GH group.

	AUC (95%CI)	p	cut-off (%)	sensitivity (95%CI)	specificity (95%CI)
**PE vs HC**					
eTreg CD297+	0.6204 (0.4325-0.8083)	0.2172	>4.445	66.67 (43.75-83.72)	50 (29.03-70.97)
exTreg CD279+	0.6466 (0.4608-0.8325)	0.1329	>9.225	66.67 (43.75-83.72)	44.44 (24.56-66.28)
FoxP3+ Helios- CD279+	0.7386 (0.5645-0.9126	0.0160	>10.12	52.94 (36.96-73.83)	88.89 (67.2-98.03)
FoxP3+ Helios+ CD279+	0.7593 (0.5978-0.9208	0.0079	>3.24	55.56 (33.72-75.44)	88.89 (67.2-98.03)
Tconv CD279+	0.6340	0.1760	>5.425	82.35 (58.97-93.81)	44.44 (24.56-66.28)
**GH vs HC**					

eTreg CD279+	0.7011 (0.5362-0.8659)	0.0323	>7.445	57.14 (36.55-75.53)	77.78 (54.79-91.0)
exTreg CD279+	0.6693 (0.4905-0.8381)	0.0714	>10.44	66.67 (45.37-82.81)	72.22 (49.13-87.5)
FoxP3+ Helios- CD279+	0.8056 (0.6560-0.9551)	0.0013	>6.075	80 (58.4-91.93)	77.78 (54.79-91.0)
FoxP3+ Helios+ CD279+	0.7963 (0.6537-0.9389)	0.0016	>2	85.71 (65.36-95.02)	66.67 (43.75-83.72)
Tconv CD279+	0.6958 (0.5280-0.8635)	0.0371	>7.410	61.9 (40.88-79.25)	72.22 (49.13-87.5)
**PE vs GH**					
eTreg CD279+	0.6296 (0.4529 - 0.8064)	0.1675	>7.155	55.56 (33.72-75.44)	57.14 (36.55-75.53)
exTreg CD279+	0.5265 (0.3412-0.7117)	0.7782	<14.05	55.56 (33.72-75.44)	52.38 (32.37-71.66)
FoxP3+ Helios- CD279+	0.6221 (0.4409-0.8032)	0.2060	<11.49	58.82 (36.01-78.39)	60 (38.66-78.12)
FoxP3+ Helios+ CD279+	0.5476 (0.3631-0.7321)	0.6121	<3.195	44.44 (24.56-66.28)	61.9 (40.88-79.25)
Tconv CD279+	0.5854 (0.4002-0.7707)	0.3706	<7.37	47.06 (26.17-69.04)	61.9 (40.88-79.25)

Table S1. Detailed results for AUC
